# HLA Class I Knockout Converts Allogeneic Primary NK Cells Into Suitable Effectors for “Off-the-Shelf” Immunotherapy

**DOI:** 10.3389/fimmu.2020.586168

**Published:** 2021-01-29

**Authors:** Keven Hoerster, Markus Uhrberg, Constanze Wiek, Peter A. Horn, Helmut Hanenberg, Stefan Heinrichs

**Affiliations:** ^1^ Institute for Transfusion Medicine, University Hospital Essen, University of Duisburg-Essen, Essen, Germany; ^2^ Institute for Transplantation Diagnostics and Cell Therapeutics, Heinrich-Heine University, Düsseldorf, Germany; ^3^ Department of Otorhinolaryngology & Head/Neck Surgery, University Hospital Düsseldorf, Heinrich Heine University, Düsseldorf, Germany; ^4^ German Cancer Consortium (DKTK), partner site Essen/Düsseldorf, Essen, Germany; ^5^ Department of Pediatrics III, University Children’s Hospital of Essen, University Duisburg-Essen, Essen, Germany

**Keywords:** NK cells, B2M knockout, HLA class I, off-the-shelf, allogeneic, genome editing, immunotherapy, adoptive cell transfer

## Abstract

Cellular immunotherapy using chimeric antigen receptors (CARs) so far has almost exclusively used autologous peripheral blood-derived T cells as immune effector cells. However, harvesting sufficient numbers of T cells is often challenging in heavily pre-treated patients with malignancies and perturbed hematopoiesis and perturbed hematopoiesis. Also, such a CAR product will always be specific for the individual patient. In contrast, NK cell infusions can be performed in non-HLA-matched settings due to the absence of alloreactivity of these innate immune cells. Still, the infused NK cells are subject to recognition and rejection by the patient’s immune system, thereby limiting their life-span *in vivo* and undermining the possibility for multiple infusions. Here, we designed genome editing and advanced lentiviral transduction protocols to render primary human NK cells unsusceptible/resistant to an allogeneic response by the recipient’s CD8^+^ T cells. After knocking-out surface expression of HLA class I molecules by targeting the B2M gene *via* CRISPR/Cas9, we also co-expressed a single-chain HLA-E molecule, thereby preventing NK cell fratricide of B2M-knockout (KO) cells *via* “missing self”-induced lysis. Importantly, these genetically engineered NK cells were functionally indistinguishable from their unmodified counterparts with regard to their phenotype and their natural cytotoxicity towards different AML cell lines. In co-culture assays, B2M-KO NK cells neither induced immune responses of allogeneic T cells nor re-activated allogeneic T cells which had been expanded/primed using irradiated PBMNCs of the respective NK cell donor. Our study demonstrates the feasibility of genome editing in primary allogeneic NK cells to diminish their recognition and killing by mismatched T cells and is an important prerequisite for using non-HLA-matched primary human NK cells as readily available, “off-the-shelf” immune effectors for a variety of immunotherapy indications in human cancer.

## Introduction

Adoptive cell transfer (ACT) of autologous genetically modified immune cells has emerged as an attractive treatment option for various malignancies of hematologic origin. Yet, the highly personalized nature of these cell products generates extreme costs and patient-specific factors can still impede the manufacturing process due to large variabilities. For a significant number of patients, an autologous final product cannot be generated in time for treatment [reviewed in (1)].

To alleviate these problems, research in the field is moving towards “off-the-shelf” products, making use of immune effector cells from healthy donors. However, this endeavor is complicated by problems of alloreactivity and immune tolerance for mismatched HLA constellations. A severe side effect of allogeneic cellular therapy is Graft-versus-Host-Disease (GvHD), a life-threatening complication caused by the transplanted alloreactive T cells and known since the early days of hematopoietic stem cell transplantation (HSCT) (2–5). To circumvent this complication, several approaches have been developed. Virus-specific cytotoxic T (VST) cells, for example, have successfully been used to control latent infections post HSCT without causing GvHD (6, 7). Accordingly, they have been proposed as a potential T cell population to create “off-the-shelf” therapeutic products (8, 9). Another option is the selective depletion of T cell subsets alloreactive towards specific HLA (10, 11). However, this bias towards certain T cell subsets is again limiting the application potential of the products. An interesting approach to abrogate unwanted or alloreactive signaling from the endogenous T cell receptors (TCRs) in chimeric antigen receptor (CAR) T cells uses genome editing on common TCR domains (12, 13), however this genomic editing will require additional gene transfer systems and therefore will add several layers of complexities to CAR T cell clinical trials.

Thus, an obvious solution is to simply use another type of immune effector cells: natural killer (NK) cells. Importantly, even when infused at large quantities into immunocompromised patients, NK cells do not cause GvHD in the first place and can even prevent it (14, 15). In contrast to T and B cells, NK cells express germline-encoded activating and inhibitory receptors and integrate signals to distinguish between healthy and transformed or stressed cells (16). This *innate* recognition of transformed cells and absence of GvHD have proven to be of great potential for the treatment of malignancies in animal models and clinical trials (17–24). While the infusion of autologous NK cells is ineffective in various cancers, donor NK cell infusions after or through haploidentical HSCT demonstrated that NK cells with killer cell immunoglobulin-like receptor (KIR) mismatches with the recipient do not cause any damage to normal tissue, but still can eliminate residual malignant cells (25–32). Importantly, mature NK cells contained within the stem cell graft were shown to be responsible for the anti-tumor effects observed early after transplantation and therefore were unlikely to originate from the reconstituted NK cell compartment (33–35). The efficacy and apparent safety of NK cells in allogeneic adoptive cell therapies has made them an attractive cell type for the manufacturing of “off-the-shelf” cell-based products. However, two major aspects of human NK cells, the marked resistance to standard genetic modifications with lentiviral vectors and the limited *ex vivo* expansion capacities, have hampered their use for both allogeneic and also autologous CAR-redirected immunotherapies. Hence, researchers have resorted to stable NK cell lines, such as NK-92 (36–42), or to using NK cells differentiated from CD34+ hematopoietic stem and progenitor cells (HSPCs) or pluripotent stem cells (PSCs) (43–46) with subsequent expansion using feeder cells (47–50).

While alloreactivity and GvHD are activities initiated by the graft, graft rejection by the host’s immune system is another factor to consider in allogeneic non-myeloablative therapies. To prevent rejection of the graft by the host immune system, a wide variety of concepts have been used, including the expression of the immune checkpoint inhibitors CTLA4-Ig and PD-L1 (51) or engagement of the “don’t eat me”-signal CD47 (52). Rather than equipping cells with means to fend off attacking immune cells, some studies arm the therapeutic cells with receptors to fight back and lyse the approaching alloreactive host T cells (9, 53). Others have set out to disrupt the HLA barrier/antigens entirely, hiding the infused cells from recognition by alloreactive host T cells. The latter aim has been achieved either by targeting genes essential for the HLA processing machinery such as the class II transcriptional activator (CIITA) and beta-2-microglobulin (B2M) or by disrupting individual HLA genes (54–67). In most of these preclinical studies however, the starting material was either a transformed cell line or PSC cells that subsequently had to be differentiated into the tissue of choice in elaborated and time-consuming protocols.

In this study, we used recent advances in genome editing and HLA biology to generate NK cells ideally suited for adoptive cellular therapy. Based on the recent breakthrough for genetically modifying human NK cells (68), we constructed a chimeric envelope with the surface and transmembrane domains of the baboon endogenous retrovirus and the cytoplasmic tail of the amphotropic murine retrovirus for efficient gene transfer, similarly to constructs described before (69–71). We then disrupted HLA class I expression in human NK cells by targeting B2M *via* a CRISPR/Cas9 lentiviral vector (72) and finally equipped the HLA class I knockout NK cells with a modified single-chain HLA-E molecule (58, 73). Consequently, these double-modified NK cells neither activated nor expanded allogeneic T cells and were also protected from autolysis/fracticide by NK cells. Combined with novel NK cell culture expansion protocols for GMP settings (74, 75), highly cytotoxic, primary “off-the-shelf” human NK cells were generated in relevant amounts without the need of lengthy differentiation protocols, PSCs or feeder cells.

## Materials and Methods

### Cells and Cell Lines

NK cells were purified from PBMC using a negative selection protocol with the NK cell isolation kit (#130-092-657, Miltenyi Biotec) and MACS LS columns (Miltenyi Biotec) according to the manufacturer’s protocol. NK cells were cultured in NK MACS medium (Miltenyi Biotec), supplemented with 1% of the enclosed NK MACS Supplement, 5% heat-inactivated human AB serum (Sigma-Aldrich, H4522), 1% Penicillin/Streptomycin (Sigma-Aldrich), 500 U/ml rhIL-2 and 140 U/ml rhIL-15 (both from Miltenyi Biotec) and termed NK MACS complete medium. T cells were purified from whole blood using the RosettaSep HLA T cell kit (Stemcell Technologies) according to the manufacturer’s protocol and cultured in DMEM (Thermo Fisher Scientific) supplemented with 5% heat-inactivated human AB serum, 1% Penicillin/Streptomycin and 50 U/ml rhIL-2 if not stated otherwise, in the following termed T cell medium. Whole blood was obtained from healthy donors at the University of Düsseldorf after informed consent. PBMNCs were isolated by Ficoll density gradient centrifugation. SKM1, K562 and Kasumi-1 cell lines where maintained in RPMI medium (Thermo Fisher Scientific) supplemented with 10% heat-inactivated FCS, termed R10 medium.

### Lentiviral Vectors

The CRISPR/Cas9 vector pLE38-Cas9-sgB2M/gNKG2A is a third-generation self-inactivating (SIN) lentiviral vector based on the pRRL SIN backbone (76). Expression cassettes for the U6-promoter/gRNA and the EFSns-promoter/Cas9 were derived from pLCv2 (72). The targeting sections of the gB2M sequence (5′-GAGTAGCGCGAGCACAGCTA-3′) and gNKG2A sequences (5′-TGAACAGGAAATAACCTATG-3′) were designed using the GPP sgRNA designer tool (Broad Institute, Cambridge, MA, USA) and cloned into the Esp3I sites of the pLE38-Cas9-stuffer vector using annealed oligonucleotides. The sc-HLA-E coding sequence was designed after Gornalusse et al. (58). Briefly, the fragment encoding the HLA-E*03:01 heavy chain was cloned from HEK293T cells with the forward-primer incorporating the last repeat of the (G_4_S)_4_ linker for the final sc-HLA-E sequence and a BamHI restriction site and the reverse-primer harboring a *MluI* restriction site for assembly into the vector. The fragment encoding the sequence for B2M-leader/HLA-G-leader/(G_4_S)_3_-linker/mutatedB2M-chain/(G_4_S)_4_-linker was synthesized by LGC genomics. The gB2M targeting site and the protospacer adjacent motif (PAM) site were mutated from 5′-CCTTAGCTGTGCTCGCGCTACTC-3′ to 5′-CACTGGCCGTGCTGGCCCTGCTG-3′ to avoid editing of the sc-HLA-E by gB2M. The HLA-G-leader-B2M-sequence was PCR amplified using primers harboring restriction sites for *Xba*I and *BamH*I and assembled together with the amplified HLA-E*03:01 heavy chain encoding fragment into a lentiviral transfer vector *via* the *Xba*I and the *Mlu*I restriction sites. Expression was driven from the SFFV promoter (77).

For construction of the pcoBaEVTM chimeric baboon envelope vector, the surface and transmembrane subunits of the wild-type sequence of the M7 strain of the Baboon endogenous virus (NC_022517) was fused to the cytoplasmic sequence of the amphotropic murine leukemia virus (AF411814), synthesized as a codon-optimized cDNA by GeneArt (ThermoFisher) according to our design and then cloned in our envelope expression plasmid using *EcoR*I and *Not*I (78).

Lentiviral particles were produced in HEK293T cells by cotransfection of pcoBaEVTM, pCMV-ΔR8.91 (76) and the lentiviral transfer vector. Supernatants were harvested 48 and 72 h after transfection, concentrated by high-speed centrifugation, resuspended in non-supplemented NK MACS medium supplemented with 20 mM HEPES and titered on K562 and SKM1.

### NK Cell Transduction

Transductions were performed 7 days after the preparation of CD56+ CD3- cells and start of the NK cell expansion protocol. Briefly, lentiviral particles corresponding to an MOI of 1 (titered on K562/SKM1) were adjusted to a volume of 100 µl using plain NK MACS medium without additives, mixed with the equal volume of plain NK MACS supplemented with 5 µg/ml Vectofusin-1 (Miltenyi Biotec), incubated at room temperature for 8 min and mixed with 50 µl cell suspension containing 1 × 10^6^ NK cells in NK MACS complete medium. For simultaneous double-transductions, both particle populations were used at MOIs of 1 and pooled prior to mixing with NK MACS and Vectofusin-1. Subsequently, the 250-µl cell/particle mix was transferred into 48-well plates and centrifuged for 90 min at 400*g*, 32°C. After spinoculation, cells were incubated at 37°C for additional 4 h before 500 µl NK MACS complete medium where carefully added to the cells.

### Flow Cytometry and Phenotyping

Flow cytometric data where acquired using a CytoFLEX (Beckman Coulter). Antibodies from Thermo Fisher Scientific were anti-HLA-E (clone 3D12; Thermo Fisher). Antibodies from BioLegend were: anti-pan-HLA class I (clone W6/32), CD3 (clone HIT3a), CD4 (clone RPA-T4), CD8 (clone SK1), CD56 (clone 5.1H11), CD16 (clone 3G8), CD57 (clone HNK-1), KIR2DL2/3 (CD158b, clone DX27), KIR2DL1/S1/3/5 (CD158a,h, clone HP-MA4), CD107a (clone H4A3), CD137 (clone 4B4-1), anti-NKG2D (CD314, clone 1D11), anti-NKp30 (clone P30-15), anti-NKp44 (clone P44-8), anti-NKp46 (clone 9E2). Antibodies from Miltenyi Biotec were anti-NKG2A (CD159a, clone REA110), anti-NKG2C (CD159c, clone REA205). Antibody stainings were performed in PBS (Thermo Fisher Scientific) supplemented with 0.5% BSA (Sigma-Aldrich) and 2 mM EDTA (Sigma-Aldrich), termed MACS buffer. Of note, the clone W6/32 does not recognize the sc-HLA-E due to the covalently linked N-terminus of the incorporated B2M (79), enabling discrimination between endogenous HLA class I and the sc-HLA-E. Analysis was performed using the CytExpert software (Beckman Coulter) and FlowJo V10.6.2 (Becton Dickinson).

### NK Cell Cytotoxicity Testing

NK cells were co-cultured for 6 h with K562 and Kasumi-1 cells in 100 µl R10 medium supplemented with 500 U/ml rhIL-2 and 140 U/ml rhIL-15 at the effector target ratios 4:1, 1:1 and 0.25:1. To allow for discrimination between NK cells and targets while avoiding to gate out dead target cells, a “no-wash” protocol was applied. Briefly, CD56-antibody was added to the wells at the end of the incubation period, mixed and stained at 4°C for 20 min. Subsequently, the cell mixture was diluted in 300 µl of MACS buffer supplemented with 7AAD and incubated for 2 min at room temperature before data acquisition. The specific lysis of targets was determined by the percentage of 7AAD^+^ cells within the CD56^−^ singlets.

### Fratricide Assay

NKG2A-KO NK cells were generated using gNKG2A, which targets the *KLRC1* gene encoding for NKG2A, and the transduction protocol as described above. The mixture of sc-HLA-E only and sc-HLA-E/B2M-KO NK cells, obtained after double transduction, was co-cultured for 24 and 48 h with either untransduced, parental NK cells from the same donor or NK cells after knockout of NKG2A. Cells were stained with 7AAD and antibodies for pan-HLA class I, HLA-E and NKG2A before acquisition. Selective depletion of B2M-KO cells was evaluated by gating on all sc-HLA-E^+^ target cells and then discriminating between HLA class I^+^ and HLA class I^−^ cells.

### T Cell Proliferation Assay

Proliferation of allogeneic T cells was evaluated by CFSE dilution. Briefly, 5 × 10^6^ freshly isolated T cells were resuspended in 5 ml prewarmed PBS/0.1% BSA. 10 µl of a 5-mM CFSE solution were added and cells were incubated at 37°C, 5% CO_2_ for 7 min, subsequently topped up with 16 ml cold DMEM/10% FCS, incubated at 4°C in the dark for 5 min and washed twice. For the assay, 200,000 T cells where co-cultured with 50,000 NK cells in 200 µl T cell medium supplemented with 2 U/ml rhIL-2 for 6 days. Cells stimulated with PMA/Ionomycin served as a qualitative positive control and medium controls were used as negative controls. On day 6, cells were stained for CD56, CD4, CD8 and 7AAD. CFSE dilution was analyzed in CD4^+^ and CD8^+^ T cells after gating on 7AAD^−^/CD56^−^ singlets.

### T Cell Reactivation and Degranulation Assay

Alloreactive T cells were expanded from isolated T cells by incubation for 14 days with 30-Gy irradiated PBMC of the respective NK cell donors. To test reactivation and degranulation of alloreactive T cells, 160,000 to 200,000 expanded T cells where incubated with 80,000 to 100,000 either parental or modified NK cells (Effector-Target ratio of 2:1) for 24 and 48 h in T cell medium. For degranulation assays, monensin and anti-CD107a antibody were added to the cultures 4 h before acquisition. For analysis, samples were stained with 7AAD and antibodies for CD3, CD4, CD8. For the reactivation assay, cells were additionally stained for CD137. Degranulation and reactivation were analyzed in CD4^+^ and CD8^+^ T cells after gating on 7AAD^−^/CD3^+^ singlets. A baseline measurement was performed on the day the assay was set up and medium, as well as autologous and 3^rd^ party NK cells, served as negative and specificity controls at the time points of analysis.

### T Cell Cytotoxicity Assay

For T vs NK cell cytotoxicity assays, CFSE-stained T cells were cultured for 20 h with the NK cell lines at the effector target ratios 4:1, 2:1 and 1:1 (calculated on CD8^+^ T cells) in 200 µl T cell medium. A “no-wash” protocol was applied to prevent loss of dead target cells: Before acquisition, the cell mixture was diluted with the same volume of MACS buffer supplemented with 7AAD and incubated at room temperature for 2 min before acquisition. Autologous NK cells served as negative controls and for gating purposes. NK cell lysis by T cells was determined as the percentage of 7AAD^+^ cells within the CFSE^−^ singlets.

### Statistics

Statistical analysis was performed using GraphPad Prism with the tests given in the figure legends. The level of statistical significance was set to p < 0.05. Statistically significant differences are reported in the figure legends.

## Results

### Concomitant Single-Chain HLA-E Expression on Primary NK Cells Allows for a Functional Knockout of HLA Class I Surface Expression Without Leading to Fratricide

**Figure 1:**
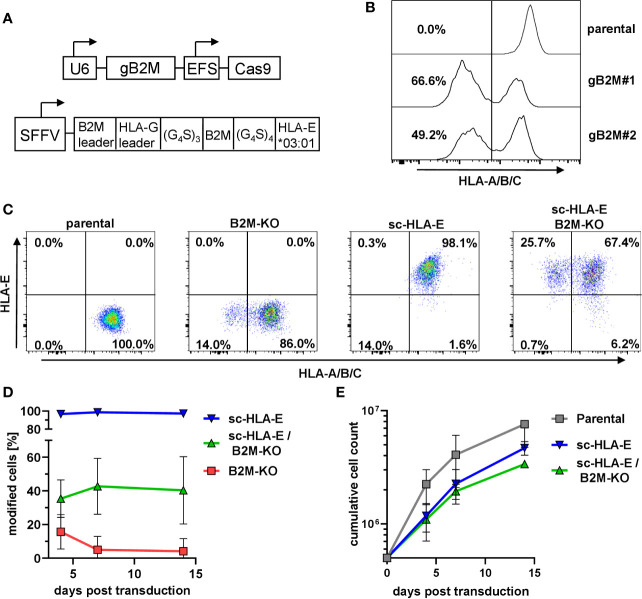
Knockout of B2M and simultaneous overexpression of sc-HLA-E in primary NK cells **(A)** Outline of the lentiviral expression cassettes used for CRISPR/Cas9 mediated B2M-knockout and expression of the sc-HLA-E molecule. Cas9 and gB2M are expressed from the same lentiviral vector, driven by an EFS and an U6 promoter, respectively. The HLA-E*03:01 heavy chain is linked to B2M and the HLA-G leader peptide by G_4_S linkers and expression is driven from an SFFV promoter. **(B)** Knockout of B2M in SKM1 cells abrogates surface expression of HLA class I detected *via* flow cytometry on day 10 after transduction. **(C)** Frequencies of HLA class I and sc-HLA-E expressing primary NK cells 4 days after transduction with lentiviral particles encoding gB2M and Cas9, sc-HLA-E or a combination of both compared to an untransduced control. **(D)** Frequencies of modified cells measured on day 4, 7 and 14 after transduction (mean ± SD; n = 6 for sc-HLA-E, n = 5 for sc-HLA-E/B2M-KO and B2M-KO). **(E)** Growth of NK cell cultures measured 4, 7 and 14 days after transduction. Shown is the cumulative cell count of all NK cells within the culture. The NK cells showed a fold-expansion from the day of transduction of 15.2 ± 5.72 for parental, 9.36 ± 1.29 for sc-HLA-E and 6.74 ± 0.31 for sc-HLA-E/B2M-KO NK cells (mean ± SD; n = 3).

In primary NK cells, the efficient knockout of the classical HLA class I genes A, B and C by CRISPR/Cas9-based genome editing is challenging due to the extensive polymorphism and the presence of six genomic target sites. The functional elimination of all HLA class I proteins with a single hit can be achieved by using a single CRISPR/Cas9 lentiviral vector targeting the beta-2-microglobulin gene (B2M), the shared invariant light chain of all HLA class I molecules (**Figure 1A**). We first tested two distinct gRNAs targeting B2M (gB2M) in the pLE38-Cas9 vector for their knockout efficiencies by transducing the human diploid AML cell line SKM1. Analyzing the transduced cells after staining with the pan-HLA class I monoclonal antibody W6/32 revealed a decrease in surface expression of classical HLA class I molecules by flow cytometry, starting four days after transduction and generating stable knockouts with both guide sequences when analyzed 10 days later (**Figure 1B**). We decided to use gB2M#1 for further experiments as it yielded a higher gene editing rate.

Despite the high gene transfer efficiencies that can be achieved in NK cells with baboon envelope-pseudotyped lentiviral vectors (68), we observed only approximately 16% HLA class I-negative cells (**Figure 1C**) four days after transduction of primary NK cells with the CRISPR/Cas9 HLA class I targeting vector. This percentage of HLA class I-negative cells steadily decreased over time and dropped below 1% on day 14 post transduction (**Figure 1D**, red line). We hypothesized that this progressive loss of successfully targeted NK cells was most likely a consequence of the “missing self”-induced killing by neighboring NK cells, a phenomenon also called *“*fratricide*”*. In parallel cultures, we therefore co-expressed a modified single-chain (sc-)HLA-E molecule (**Figure 1A**) on the surface of NK cells, as this chimeric protein can efficiently protect the HLA class I-negative NK cells from fratricide by engaging the inhibitory receptor dimer CD94/NKG2A (58). By itself, lentiviral overexpression of sc-HLA-E yielded a distinct positive population that was stable over time (**Figures 1C, D**, blue line). By pooling the two lentiviral supernatants encoding sc-HLA-E and sgB2M/Cas9, we achieved a mean gene editing/transduction frequency of 35.4% that remained stable around 40.4% on days 7 and 14 post transduction (**Figures 1C, D**, green line). NK cell expansion was documented for 14 days after transduction (3 weeks after isolation), demonstrating that sc-HLA-E and sc-HLA-E/B2M-KO modified NK cells indeed grew slower compared to the mock-transduced controls (“parental”), but still achieved an almost seven-fold expansion from the day of transduction (**Figure 1E**). NK cells were used for downstream experiments within 4 weeks from the day of preparation.

### Protection From Fratricide Is Dependent on NKG2A

**Figure 2:**
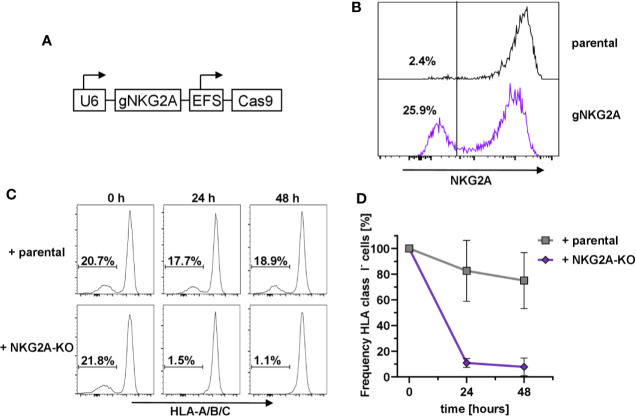
Dependency on NKG2A expression for sc-HLA-E-mediated protection from NK cell fratricide **(A)** Outline of the lentiviral expression cassettes used for CRISPR/Cas9 mediated NKG2A-knockout. **(B)** Transduction with Cas9 and gNKG2A led to abrogation of NKG2A surface expression in primary NK cells 7 days post transduction. **(C)** Representative histograms showing the frequency of sc-HLA-E/B2M-KO NK cells in presence of B2M-competent sc-HLA-E NK cells upon addition of parental cells (upper panel) or NKG2A-KO NK cells (lower panel) at different time points. **(D)** Quantification of changes in sc-HLA-E/B2M-KO NK cell frequencies relative to the time point (0 h) when parental or NKG2A-KO NK cells were added. Statistical analysis was performed by two-way ANOVA with Geisser-Greenhouse correction (ε = 0.6972) for repeated measures and Holm-Sidak testing for multiple comparisons between each time point within both groups. For the samples containing NKG2A-KO NK cells, the decrease in frequency of HLA class I^−^ was statistically significant at both time points (mean ± SD; n = 4).

In order to prove that the HLA-E-mediated protection of HLA class I-negative NK cells in mixed cultures was due to the engagement of the inhibitory receptor CD94/NKG2A (73), we designed the corresponding guide RNAs against *KLRC1*, the gene encoding NKG2A, and used the same lentiviral expression system as above (**Figure 2A**). Flow cytometry analysis demonstrated that transduction of primary human NK cells with the pLE38-Cas9 vector successfully abrogated NKG2A expression seven days post transduction (**Figure 2B**). To test whether *NKG2A*-KO NK cells lead to the relative reduction of sc-HLA-E/B2M-KO NK cells, as they would no longer be tolerated by NKG2A-deficient NK cells, the bulk-transduced NKG2A-KO NK cell cultures were co-cultured with a mixture of sc-HLA-E/B2M-KO and sc-HLA-E NK cells. Importantly, addition of NKG2A expressing parental NK cells did not have any effect on the frequency of sc-HLA-E/B2M-KO NK cells in a mix with B2M competent sc-HLA-E-expressing NK cells when compared to the baseline controls (**Figure 2C**, upper panel and **Figure 2D**). In contrast, a co-culture containing NKG2A deficient NK cells showed a strong depletion of the sc-HLA-E/B2M-KO NK cells after 24 h, leading to elimination of more than 85% of B2M-KO cells (**Figure 2C**, lower panel and **Figure 2D**). In support of these observations, we also noted that B2M-KO NK cells did not persist in NK cell cultures with low NKG2A-expression levels despite the presence of sc-HLA-E (data not shown). These results demonstrated that the prevention of fratricide is strongly dependent on the HLA-E/NKG2A signaling.

### B2M-KO NK Cells Are Phenotypically Similar to Unmodified Cells and Retain Uncompromised Effector Functions

**Figure 3:**
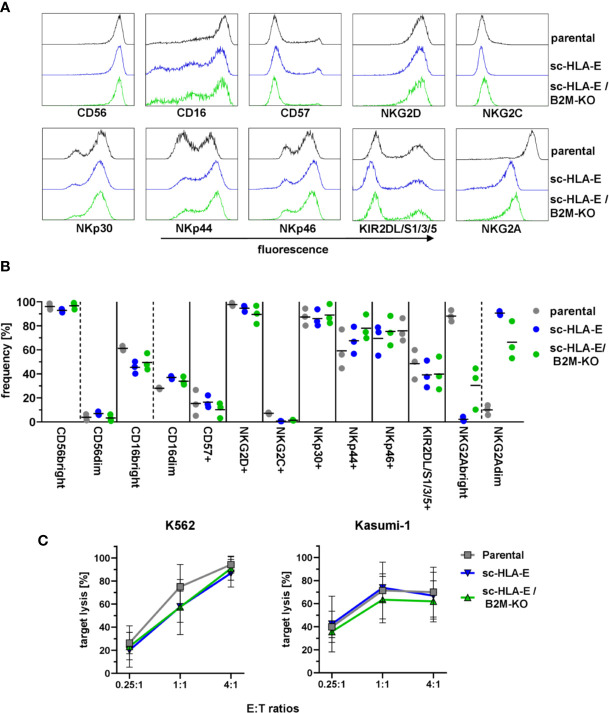
Functional and phenotypical analysis of the gene modified NK cells. **(A)** Representative flow cytometry plots of the phenotypical analysis of the NK cell cultures. **(B)** Quantification of NK cell frequencies for phenotypic markers in parental, sc-HLA-E and sc-HLA-E/B2M-KO NK cell cultures from left to right for each marker (all data points shown, n = 3). **(C)** Quantification of natural NK cell cytotoxicity by lysis of the AML cell lines K562 and Kasumi-1 after co-incubation for 6 h. Statistical analysis was performed by two-way ANOVA with Holm-Sidak testing for multiple comparisons. No statistically significant differences could be detected (mean ± SD; K562: n = 6 for parental and sc-HLA-E/B2M-KO and n = 5 for sc-HLA-E NK cells; Kasumi: n = 4 for parental and sc-HLA-E/B2M-KO and n = 3 for sc-HLA-E NK cells).

HLA molecules serve a vital role in the education of NK cells *via* KIRs and NKG2A (80, 81). As the forced expression of HLA-E on neighboring cells might lead to tonic engagement of CD94/NKG2A, which can signal *via* downstream targets (82, 83), we next investigated if the genetic modifications in HLA expression impacted the NK cell phenotype and functions. To this end, we performed multi-color flow cytometric analysis using a panel comprising various maturation markers as well as activating and inhibitory receptors. The results in **Figures 3A, B** demonstrated that the modified NK cells exhibited an immunophenotypic profile similar to their unmodified counterparts: The majority of cells was CD56^bright^ and CD16 was expressed on almost all cells with a slight bias towards CD16^bright^ cells. Only a minor fraction of NK cells expressed CD57 usually associated with terminal maturation and replicative senescence (84), while almost all cultured NK cells were positive for the activating receptors NKG2D and NKp30. NKp44 and NKp46 was present on about 60 – 80% of NK cells from three donors, but little to no NKG2C^+^ cells were detected. KIR2DL/S1/2/3 expression was detectable on roughly 50% to 60% of NK cells while NKG2A expression was present in over 90% of cells. Interestingly, the expression levels of NKG2A were diminished in both sc-HLA-E-expressing NK cells, rendering these cells NKG2A^dim^. In addition, the frequencies of CD16, NKG2C and KIR expressing NK cells were slightly lower in the sc-HLA-E-expressing NK cell cultures compared to parental NK cells while the frequency of NKp44 expressing NK cells in cultures expressing sc-HLA-E was slightly higher.

To test the cytotoxic effector cell functions, the genetically modified NK cells were co-incubated with the AML cell lines K562 (HLA class I^−^) and Kasumi-1 (HLA class I^+^). Flow cytometric analysis after 6 h of co-incubation revealed uncompromised natural cytotoxicity towards both AML cell lines in a dose-dependent fashion with no statistically significant differences detectable (**Figure 3C**). Therefore, the high cytotoxicity towards the HLA class I^+^ Kasumi-1 cells highlighted that the remarkable cytotoxicity of the NK cells against AML blasts is not inhibited by the genetic modifications using either CRISPR/Cas9 technology or lentiviral overexpression of HLA molecules.

### Expression of sc-HLA-E Suppresses Proliferation of Allogeneic T Cells

**Figure 4:**
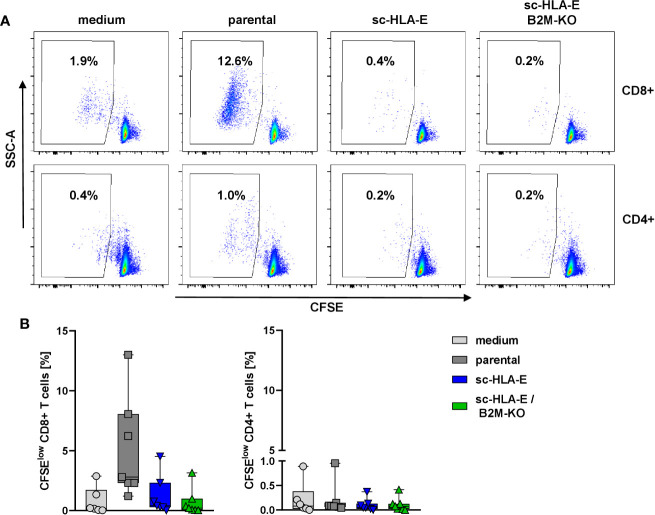
CFSE proliferation assay to measure the immune response of allogeneic T cells towards the modified NK cells. **(A)** Representative flow plots showing the frequency of CFSE^low^ CD8^+^ (upper panel) and CD4^+^ (lower panel) T cells after 6 days of co-incubation with NK cells carrying the modifications depicted above the plots. **(B)** Quantification of activated CFSE^low^ CD8^+^ (left) and CD4^+^ (right) T cells after co-incubation with the modified NK cells. Statistical analysis was performed using one-way ANOVA with Holm-Sidak testing for multiple comparisons. The higher frequency of CFSE^low^ CD8^+^ T cells was statistically significant compared to sc-HLA-E and sc-HLA-E/B2M-KO NK cells (Box plots including median, quartiles and all data points, n = 7).

In the next set of experiments, we wanted to explore whether the modifications of HLA class I surface expression also conferred escape of immune recognition by allogeneic T cells. An allogeneic T cell response can be initiated *via* two different pathways, either a direct recognition by binding of the TCR to the foreign HLA proteins themselves or indirectly by donor peptides presented on self-HLA molecules by antigen-presenting cells (85). Through both pathways, T cells become activated, exert effector functions and undergo clonogenic expansion. We therefore measured the expansion of allogeneic T cells from healthy unrelated donors as a surrogate for immune recognition in a mixed lymphocyte culture (MLC) with the modified NK cells in comparison to parental NK cells and a medium control. To this end, purified T cells were labeled with the dye CFSE and co-cultured with NK cells. After six days, the proliferation of the T cells was assessed by flow cytometry as the frequency of CFSE^dim^ cells. The data in **Figure 4** demonstrated that the CD4^+^ T cells did not proliferate in response to HLA mismatched NK cells irrespectable whether these cells overexpressed sc-HLA-E or not and also independent of the B2M status. In contrast, co-culture with parental unmodified NK cells activated alloreactive CD8^+^ T cells and induced their proliferation, visible as the increased percentage of CFSE^dim^ cells (**Figure 4A** second panel top row and **Figure 4B** first panel). Surprisingly, sc-HLA-E only NK cells also did not induce allogeneic CD8^+^ T cell proliferation, despite intact HLA class I expression (**Figure 4A**, lower panel). Quantification showed that, while allogeneic responses vary greatly between the individual pairs in the mixed MLCs, a significant allogeneic stimulus was only generated by unmodified NK cells and only for CD8^+^ T cells (**Figure 4B**).

### Only B2M-KO NK Cells Are Protected From Allogeneic CD8^+^ T Cell Responses

As only unmodified allogeneic NK cells elicited a proliferative response in CD8^+^ T cells, we hypothesized that the overexpression of sc-HLA-E can actively suppress T cell activation/proliferation and consequently cytotoxicity even after direct TCR-mediated recognition of the foreign HLA on the target NK cells. Therefore, in order to investigate whether mere overexpression of sc-HLA-E in NK cells is sufficient to protect them from alloreactive T cell cytotoxicty, we evaluated T cell degranulation and subsequent lysis of parental, sc-HLA-E or scHLA-E/B2M-KO NK cells by HLA-mismatched T cells. As only a fraction of T cells is capable of directly recognizing foreign HLA molecules for any given donor-recipient pair, expansion of the alloreactive T cells occured prior to the experiments by co-culture (“priming”) with 30 Gy-irradiated PBMCs of the specific NK cell donor for 14 days. Subsequently, these T cells were co-cultured with NK cells and then analyzed for expression of CD137 or CD107a as markers for activation and degranulation, respectively. Autologous and also HLA-disparate “3^rd^ party” NK cells served as important controls.

**Figure 5:**
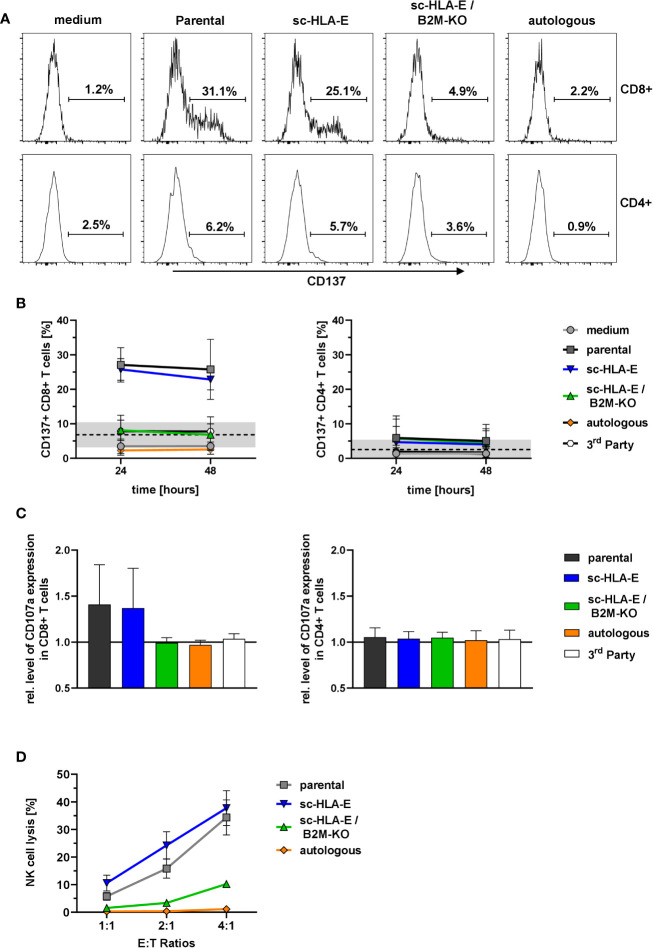
Activation and degranulation of primed T cells in presence of modified NK cells. **(A)** Representative flow plots showing the frequency of CD137^+^ cells within CD8^+^ (upper panel) and CD4^+^ (lower panel) T cell subsets after 24 h of co-incubation with the different NK cell cultures depicted above the plots. **(B)** Quantification of CD137^+^ frequencies among CD8^+^ (left) and CD4^+^ (right) T cells at two time points. Statistical analysis was performed by two-way ANOVA with matching by time points and Holm-Sidak testing. At both time points, the frequency of CD137^+^ cells within the CD8^+^ T cells was significantly lower when challenged with sc-HLA-E/B2M-KO NK compared to parental and sc-HLA-E NK cell containing cultures (mean ± SD; n = 7 for medium control, parental, sc-HLA-E, sc-HLA-E/B2M-KO NK cells, n = 4 for autologous and 3^rd^ party controls; black line and grey box indicate the mean of the baseline measurements ± 95% confidence interval). **(C)** Levels of CD107a normalized to the medium controls in CD8^+^ (left) and CD4^+^ (right) T cells 24 h after co-incubation with NK cells. Statistical analysis was performed using Friedman test with Dunn’s correction for multiple comparisons. Levels of CD107a were significantly lower for CD8^+^ T cells incubated with sc-HLA-E/B2M-KO NK cells compared to T cells incubated with parental or sc-HLA-E NK cells (mean ± SD; n = 7 for medium control, parental, sc-HLA-E, sc-HLA-E/B2M-KO NK cells, n = 4 for autologous and 3^rd^ party controls). **(D)** Lysis of NK cells by primed T cells at the effector targets ratios 4:1, 2:1 and 1:1 (calculated on CD8^+^ T cells) after 20 h of co-incubation. Statistical analysis was performed by two-way ANOVA with Holm-Sidak testing for multiple comparisons. The reduced lysis of sc-HLA-E/B2M-KO NK cells was statistically significant at E:T ratio 4:1 compared to parental and sc-HLA-E NK cells and at ratio 1:1 compared to sc-HLA-E NK cells but not to parental NK cells, yet the p value almost met the criterion with p = 0.0516 (mean ± SD; n = 5).

These co-culture experiments with primed T cells revealed a specific activation of CD8^+^ but not CD4^+^ T cells in presence of parental as well as sc-HLA-E-expressing NK cells for up to 48 h as measured by CD137 expression (**Figures 5A, B**). In contrast, sc-HLA-E/B2M-KO NK cells did not induce expression of CD137 in a significant fraction of CD8^+^ T cells, similarly to co-cultures with autologous and also 3^rd^ party NK cells, thus confirming the donor specificity of the assay. Analysis of degranulation by CD107a staining at 24 h of co-culture revealed a similar pattern (**Figure 5C**), with degranulation in the presence of parental and sc-HLA-E-expressing NK cells, while the CD107a levels of T cells challenged with sc-HLA-E/B2M-KO NK cells was comparable to those using autologous and 3^rd^ party controls. Finally, the specific cytotoxicity towards the different genetically modified NK cells was assessed with purified populations at effector to target ratios of 4:1, 2:1 and 1:1 (calculated on CD8^+^ T cells). After 20 h of co-culture with primed T cells (**Figure 5D**), between 10 and 40% of parental and sc-HLA-E expressing NK cells were killed in a dose-dependent manner. In contrast, sc-HLA-E/B2M-KO NK cells were not lysed at E:T ratios of 1:1 and 2:1, and even E:T ratios of 4:1 resulted in only <10% lysis.

## Discussion

In this study, we have established a robust methodology to generate primary NK cells that are devoid of classical HLA class I molecule surface expression. Compared to their unmodified counterparts, the genome-edited NK cells escaped immune recognition by mismatched CD8^+^ and CD4^+^ T cells, thus making them suitable tools for “off-the-shelf” allogeneic immunotherapy. To achieve this, we first had to overcome the obstacle that NK cells are “hard-to-transduce” cells. This *relative* resistance of primary human NK cells to lentiviral and also alpha-retroviral vectors using VSV-G or RD114 pseudotypes was just recently documented again (86) and is simply due to the low expression levels of the cellular proteins that serve as surface receptors for entry of such pseudotyped vector particles (68). Based on the pioneering work of Els Verhoeyen and her colleagues establishing the envelope of the baboon endogenous virus (BaEV) as novel pseudotype for human primary cells (69), two recent studies demonstrated efficient NK cell transduction with the BaEVRless envelope using either CH296/retronectin-coated plates (87) or Vectofusin-1 as enhancers of viral uptake (68, 69, 88). However, as the BaEVRless envelope with the deletion of the R protein is highly fusogenic already in the packaging cells, we constructed another version of the BaEV envelope featuring a fusion of the surface and transmembrane regions with the cytoplasmic tail of the amphotropic endogenous murine retrovirus, as described (69). This pseudotype for lentiviral vectors enabled us to reproducibly and efficiently perform genome editing of primary NK cells.

In past clinical trials, mainly genetically non-manipulated allogeneic NK cells were used for immunotherapy of malignancies including AML, myeloma and solid tumors (17, 20, 22, 23, 89). The clinical response rates were highly variable, ranging from 26 to 50% and often with only transient improvements (17, 20, 22, 23). Remarkably, no GvHD was observed in these trials despite the various HLA mismatch constellations, except for one study with higher T cell contaminations (89). All NK cell trials had two things in common: (i) preconditioning therapy using fludarabine and cyclophosphamide to deplete recipient lymphocytes in order to avert immunological rejection, and (ii) subcutaneous injections of IL-2 to facilitate NK cell engraftment and maintenance. The study from Miller et al. (20) showed that only high-intensity conditioning using fludarabine and high-dose cyclophosphamide was able to facilitate engraftment of NK cells beyond day 5 post infusion, compared to regimens that administered only fludarabine or low-dose cyclophosphamide and prednisolone, arguing that rigorous lymphodepletion is indispensable for successful engraftment and post-injection expansion. Importantly, the lymphodepleting conditioning was accompanied by a rise of endogenous IL-15 levels which roughly correlated with NK cell *in vivo* expansion. In all trials with high-dose conditioning (17, 20, 22, 23), donor-derived NK cells were detected in the patients by PCR for up to 28 days post infusion. During these four weeks, a decline in numbers was usually evident between 8 and 17 days (17, 20, 22, 23), which coincided with the patients’ with hematopoietic recovery and rise in endogenous T cell counts. Additionally, Shi et al. (23) reported that T cells from patients treated with NK cells showed reactivity towards donor-derived PBMNCs in an *in vitro* MLR. This finding is bolstered by the observation by Curti et al. (17) that a second infusion of NK cells is rejected even quicker than the first one: 5 days vs. 17 days, respectively. Taken together, these results strongly suggest that the mounting of an alloreactive immunological T cell memory response is a major contributing factor for the short-term NK cell persistence. Shi and colleagues even argued that the regular IL-2 injections might have facilitated the quick establishment of an allogeneic T cell response (23).

Thus, these clinical studies highlight the potential benefit of a knockout of HLA class I for allogeneic NK cell therapy to avoid donor-specific alloreactions of the patient’s T cells and extend the persistence of the transfused NK cells. In addition, the evasion from a pool of alloreactive patient T cells, whose numbers would inevitably build up due to indirect allorecognition after infusion, should readily enable multiple infusions and even has the potential to make lymphotoxic conditioning obsolete.

We achieved the functional deficiency of HLA class I molecules by a lentiviral CRISPR/Cas9-mediated knockout of B2M. However, given that HLA class I expression protects against NK cell recognition, it is not surprising that B2M-KO NK cells did not persist in culture, but were lysed by their neighboring NK cells based on the “missing self” activation. The phenomenon of NK cells killing each other, called fratricide, has been observed before, yet in other contexts. In murine NK cells, for example, trogocytosis of NKG2D ligands from tumor cells can trigger fratricide, which has been proposed as a negative feedback loop to control NK cell activation (90). Patients with multiple myeloma, who were treated with the monoclonal antibody daratumumab targeting CD38, clinically benefitted from the antibody treatment. However, an unexpected side effect was the loss of CD38+ autologous NK cells in the peripheral blood and even in the bone marrow of the patients *via* an antibody-dependent cellular cytotoxicity (ADCC) (91, 92). In an experimental setting, this fratricide of autologous CD38+ NK cells was overcome by a CRISPR/Cas9-mediated knockout of CD38 in *in vitro* expanded NK cells (91), thus providing a potential therapeutic strategy to enhance the efficacy of the antibody infusions further.

To avoid fratricide, we co-expressed a sc-HLA-E molecule as described by Gornalusse and colleagues (58) as an efficient approach to protect HLA-deficient PSC-derived cells from NK cell lysis. Despite the necessity to introduce two genetic modifications in the NK cells, the knockout of B2M and the overexpression of sc-HLA-E, we noted only a minor reduction in the expansion kinetics/characteristics of our NK cell cultures, when we transduced the cells simultaneously with the mixture of both concentrated supernatants. It seems likely that this reduction can be attributed to the higher vector doses used to achieve efficient transduction and editing frequencies. For clinical purposes however, the NK cells will be expanded for at least 21 days in a closed system such as the Prodigy (74, 75), thus sufficient opportunities for sequential genetic manipulations can be established in an optimal cell expansion protocol. Additionally, there is no need to purify the edited cells, as they would simply persist due to their immune evasive properties, thus facilitating a simple manufacturing process. To further validate the fratricide hypothesis and exclude that the loss of B2M directly led to NK cell death, we performed the fratricide assays using NK cells in which the *KLRC1* gene, coding for the inhibitory receptor NKG2A that recognizes sc-HLA-E, had been knocked out by genome editing. In these experiments, NKG2A-deficient NK cells eliminated the B2M-KO cells, regardless of whether sc-HLA-E was expressed or not.

The phenotypical and functional analyses revealed robust concordances between the parental and the genetically modified NK cells. While the killing of established target cells for NK cells such as K562 was comparable, the only notable difference between the parental NK cells and those expressing sc-HLA-E, regardless of the B2M-KO, was the lower expression level of NKG2A. One obvious explanation of the diminished NKG2A surface expression here is that the overexpressed sc-HLA-E already binds to NGK2A within the cells, thus leading to retention of the complex. This idea is clearly reminiscent of the approach developed by Kamiya and colleagues, in which they engineered NKG2A^dim/−^ NK cells for immunotherapy by cytoplasmically targeting NKG2A with a scFv fused to an endoplasmatic reticulum (ER) retention peptide, thereby retaining NKG2A in the ER (93).

Curiously, sc-HLA-E-expressing NK cells with intact HLA class I expression did not evoke allogeneic T cell proliferation, while the sc-HLA-E positive NK cells were still efficiently lysed when the same T cells were pre-activated in an MLR with irradiated feeder cells for 14 days. One obvious explanation is that the frequency of alloreactive T cells against a specific HLA type is relatively low and that these few T cells upon activation upregulate NKG2A. The newly expressed NKG2A is rapidly engaged by a sc-HLA-E molecule on neighboring NK cells, thus hampering the activation and the proliferation of the alloreactive T cells. Although this situation can readily occur in an *in vitro* setting in which the activated T cell is surrounded by sc-HLA-E expressing NK cells, *in vivo* the likelihood of such interactions is very low and one can expect numerous events of indirect immune recognition that will inevitably generate a large pool of alloreactive T cells capable of eliminating all HLA divergent cells.

In summary, we think that the universal “off-the-shelf” effector cell product for adoptive cellular therapies should be B2M-deficient NK cells overexpressing sc-HLA-E. These cells will be completely invisible for allogeneic T cell responses and will be protected from NKG2A+ recipient NK cells. Whether these modifications are sufficient for such modified NK cells to evade recognition and destruction of the patient’s immune system needs to be explored in clinical trials. Nevertheless, our modifications appear to be highly valuable to enhance efficacy of CAR-modified NK cells. Indeed, a recently published seminal NK cell study for CD19-positive lymphoid tumors by Liu and colleagues used a single dose of partly matched (mostly 4/6 with regard to A, B and DRβ1) allogeneic NK cells that had been transduced with a retroviral vector encoding three different transgenes: a CD19 CAR, soluble IL-15 and the iCASP9 suicide gene (19). In eleven treated patients, neither GvHD nor a cytokine release syndrome occurred. Thus, the suicide gene was never employed (19). Independent of the cell doses infused, eight patients (75%) had a clear immune response against the CD19+ malignant cells, which was complete and lasting in seven out of the eight patients. Remarkably, the additionally expressed IL-15 appeared to promote the long-term expansion of the donor NK cells *in vivo* for up to 12 months (19). Although cellular alloreactions by the recipients’ T cell systems subsequent to the infusions were not tested and probably strongly influenced by the lymphodepleting conditioning in these heavily pretreated patients, it cannot be ruled out that the high degree of HLA matching, the expression of IL-15 and the variety of additional treatments and substances that the patients received after the NK cell infusions all played major roles.

Our study adds the knockout of B2M in combination with sc-HLA-E expression as another building block to the development of “off-the-shelf” cellular NK cell therapies to enable manufacturing of safer and more efficient cell products to benefit a larger group of patients.

## Data Availability Statement

The raw data supporting the conclusions of this article will be made available by the authors, without undue reservation.

## Author Contributions

KH and SH conceived the experiments. KH and CW performed experiments and analyzed data. KH, MU, HH, PH and SH wrote the manuscript. All authors contributed to the article and approved the submitted version.

## Funding

This work was conducted in the framework of the iCAN33 project, funded by the European Regional Development Fund NRW (ERDF, German EFRE) 2014-2020. We acknowledge support by the Open Access Publication Fund of the University of Duisburg-Essen.

## Conflict of Interest

The authors declare that the research was conducted in the absence of any commercial or financial relationships that could be construed as a potential conflict of interest.
